# Autonomic arousal detection and cardio-respiratory sleep staging improve the accuracy of home sleep apnea tests

**DOI:** 10.3389/fphys.2023.1254679

**Published:** 2023-08-24

**Authors:** Marco Ross, Pedro Fonseca, Sebastiaan Overeem, Ray Vasko, Andreas Cerny, Edmund Shaw, Peter Anderer

**Affiliations:** ^1^ Philips Sleep and Respiratory Care, Vienna, Austria; ^2^ Department of Electrical Engineering, Eindhoven University of Technology, Eindhoven, Netherlands; ^3^ Philips Research, Eindhoven, Netherlands; ^4^ Sleep Medicine Center Kempenhaeghe, Heeze, Netherlands; ^5^ Philips Sleep and Respiratory Care, Pittsburgh, PA, United States

**Keywords:** polygraphy, polysomnography, home sleep apnea test, autonomic arousals, cardio-respiratory sleep staging, apnea-hypopnea index, artificial intelligence

## Abstract

**Introduction:** The apnea-hypopnea index (AHI), defined as the number of apneas and hypopneas per hour of sleep, is still used as an important index to assess sleep disordered breathing (SDB) severity, where hypopneas are confirmed by the presence of an oxygen desaturation or an arousal. Ambulatory polygraphy without neurological signals, often referred to as home sleep apnea testing (HSAT), can potentially underestimate the severity of sleep disordered breathing (SDB) as sleep and arousals are not assessed. We aim to improve the diagnostic accuracy of HSATs by extracting surrogate sleep and arousal information derived from autonomic nervous system activity with artificial intelligence.

**Methods:** We used polysomnographic (PSG) recordings from 245 subjects (148 with simultaneously recorded HSATs) to develop and validate a new algorithm to detect autonomic arousals using artificial intelligence. A clinically validated auto-scoring algorithm (Somnolyzer) scored respiratory events, cortical arousals, and sleep stages in PSGs, and provided respiratory events and sleep stages from cardio-respiratory signals in HSATs. In a four-fold cross validation of the newly developed algorithm, we evaluated the accuracy of the estimated arousal index and HSAT-derived surrogates for the AHI.

**Results:** The agreement between the autonomic and cortical arousal index was moderate to good with an intraclass correlation coefficient of 0.73. When using thresholds of 5, 15, and 30 to categorize SDB into none, mild, moderate, and severe, the addition of sleep and arousal information significantly improved the classification accuracy from 70.2% (Cohen’s *κ* = 0.58) to 80.4% (*κ* = 0.72), with a significant reduction of patients where the severity category was underestimated from 18.8% to 7.3%.

**Discussion:** Extracting sleep and arousal information from autonomic nervous system activity can improve the diagnostic accuracy of HSATs by significantly reducing the probability of underestimating SDB severity without compromising specificity.

## 1 Introduction

Polysomnography (PSG) is the gold standard to assess sleep as well as associated events. Polygraphy (PG) refers to a recording setup without neurological signals, often limited to respiration, pulse-oximetry, and body movements, and is an often-used tool for confirming suspected obstructive sleep apnea (OSA) in adults (Rosen et al., 2018). These ambulatory devices are often referred to as home sleep apnea tests (HSATs). Studies comparing HSATs to simultaneously recorded in-lab PSG suggest that these polygraphic devices can achieve good performance for the categorization of sleep disordered breathing (SDB) severity, especially when compared to the interscorer variability amongst manual experts scoring PSG. A systematic review of 59 studies evaluating the diagnostic accuracy of portable sleep tests without neurological signals compared to polysomnography including neurological channels concluded that HSATs are an appropriate diagnostic tool for adults with a high pretest probability of moderate to severe OSA (El Shayeb et al., 2014). However, they also highlight that the severity of mild and moderate SDB might be underestimated, which may impact the subsequent treatment plan (Bianchi and Goparaju, 2017; Massie et al., 2018; Van Pee et al., 2022).

The apnea-hypopnea index (AHI), calculated as the number of apneas and hypopneas per hour of sleep, is the primary measure for quantifying SDB severity in clinical practice. Diagnostic thresholds of 5, 15, and 30 events per hour are recommended to discriminate between no, mild, moderate, and severe OSA (Epstein et al., 2009). In full PSGs, the AHI can be considered to be a very robust measure with high inter-scorer agreement, with reported intraclass correlation coefficients (ICCs) across independent manual scorings between 0.84 and 1.00, depending on the applied hypopnea scoring rules (Kuna et al., 2013; Magalang et al., 2013; Malhotra et al., 2013; Punjabi et al., 2015; Massie et al., 2018; Van Pee et al., 2022). Historically, different hypopnea scoring criteria have been defined, requiring flow amplitude reductions of 30 or 50 percent, confirmation by 3% or 4% relative desaturations, confirmation with or without arousals, and sometimes even without any additional confirmation if the amplitude reduction was at least 50 percent (AASM, 1999; Iber et al., 2007; Berry et al., 2012). The impact of applying different hypopnea scoring rules on the diagnostic outcome has been studied in previous work, suggesting that using arousal events for the scoring of hypopneas facilitates the identification and treatment of a wider spectrum of symptomatic patients without a significant loss of scoring reliability (Ruehland et al., 2009; Berry et al., 2012; Anderer et al., 2022). Therefore, in 2012, the American Academy of Sleep Medicine (AASM) revised the recommended scoring rule for hypopneas in PSG recordings of adults: a respiratory event shall be scored as hypopnea if there is a 30% reduction in peak signal excursions from the pre-event baseline in the respiratory flow signal for at least 10 s and there is a ≥3% oxygen desaturation from pre-event baseline or the event is associated with an arousal (Berry et al., 2012). This limits the applicability of the rule to PSG recordings because neurological signals, required to score cortical arousals, are typically not collected during HSATs. In fact, the crucial role of cortical arousals (to confirm hypopneas) and of sleep scoring (to obtain total sleep time required to calculate AHI) underpins the limited capability of HSATs to assess mild and moderate SDB.

Recently, artificial intelligence-based classifiers achieved remarkable results in classifying sleep from cardio-respiratory signals like photoplethysmography (PPG) and respiratory effort/flow or peripheral-arterial tonometry (PAT), effectively enabling the scoring of sleep in HSAT recordings without neurological signals (Hedner et al., 2011; Beattie et al., 2017; Li et al., 2018; Radha et al., 2019; Fonseca et al., 2020; Sun et al., 2020; Bakker et al., 2021). Although these algorithms do not yet reach the accuracy of sleep staging using neurological signals, it has been shown that they can provide accurate estimates of the total sleep time as well as of REM sleep periods. It thus follows that these surrogate estimations help improve the diagnostic sensitivity of HSATs for detecting sleep disordered breathing and can even enable the detection of REM-related OSA (Bakker et al., 2021; Massie et al., 2022). Similarly, advances were made in the detection of autonomic arousals in HSATs as a surrogate for cortical arousals. Using PAT, or electrocardiography, good agreement in the detection of arousals is achieved (Pillar et al., 2002; Olsen et al., 2018; Li et al., 2020). A first device that uses autonomic arousals to aid the detection of respiratory events is already available (Massie et al., 2018).

In this study, we evaluated the effect of applying the recommended scoring rules for respiratory events as defined for PSG, but based on signals acquired solely with HSATs instead. We used a previously developed cardio-respiratory sleep staging algorithm to obtain a surrogate measure of total sleep time and developed a new artificial neural network to detect autonomic arousals from photoplethysmography (PPG) and respiratory flow. We evaluated the performance of our autonomic arousal detection for estimating the arousal index and the AHI estimation as compared to gold standard PSG. Subsequently, we assessed the impact of adding sleep and arousal estimation on the SDB severity classification performance in polygraphic recordings.

## 2 Materials and methods

### 2.1 Datasets

To analyze the effect of autonomic arousal detection on the diagnostic accuracy of HSATs, acquisitions from two databases were combined to train an artificial neural network to detect autonomic arousals.

The first database, called Somnoval, has been previously used to validate the Somnolyzer auto-scoring system (Punjabi et al., 2015). It consists of 97 routine in-lab PSG recordings of patients referred to three different sleep laboratories. Each PSG recording was scored independently for sleep stages, arousals, and respiratory events by four registered polysomnographic technologists (RPSGTs) according to the version 1.0 of the AASM scoring guidelines. The data consisted of approximately one-third diagnostic studies, one-third positive airway pressure (PAP) titration studies, and one-third split nights where the first portion of the recording contains a diagnostic montage confirming OSA while the second portion of the night is used for PAP titration. The study protocol was approved by the institutional review board of each clinical site. We artificially created HSAT recordings by copying a limited set of channels that would typically be available in a HSAT (respiratory flow, thoracic effort, SpO2, snoring, pulse rate, body position, raw PPG). Only one flow signal was retained when creating the reduced (HSAT) versions of the PSG studies: the nasal pressure flow in diagnostic nights and for the first portion of split nights, and the treatment device flow during titration nights and the second portion of split nights.

The second database, called Somnapatch, consisted of 190 acquisitions with full PSG and simultaneously recorded HSAT using a shared nasal canula. We used the pressure flow signals sampled at 100 Hz for exact time-alignment of the parallel recordings, correcting for clock shift and clock drift using cross-correlation between the two recordings in moving windows. We excluded all recordings where the quality of one or both pressure flow signals was too low to reliably determine the clock shift and clock drift for the entire recording. Twelve acquisitions were excluded due to missing data or bad data quality and another thirty recordings were excluded because the time-alignment between the PSG and the HSAT recordings was not reliably possible, resulting in a total number of 148 recordings from this database that were used in the present study. Informed consent was obtained during the first visit of each participant, prior to the monitoring night.

### 2.2 Scoring of PSG recordings

To obtain ground-truth scorings following the latest standards, sleep stages, cortical arousals, and respiratory events were scored with the Somnolyzer auto-scoring system embedded as an optional component in Sleepware G3 version 4.0.2.0 for all PSG recordings according to the sleep and sleep disordered breathing scoring rules recommended in the AASM manual version 2.6 (Berry et al., 2020). Somnolyzer has recently been validated and shown to be non-inferior to manual expert scoring regarding sleep stages (Bakker et al., 2023), as well as the apnea-hypopnea index (AHI) and the arousal index (ArI) (Anderer et al., 2022). Therefore, the Somnolyzer auto-scoring system has been cleared by the U.S. Food & Drug administration to be used with adults to generate an output that is ready for review and interpretation by a physician (510K number: K202142). Hypopneas were confirmed by three percent relative oxygen desaturations and/or cortical arousals. In full PSG recordings, sleep stages and arousals were derived from neurological signals using all frontal, central, occipital EEG, both EOG, and the chin EMG channels. We determined the reference (cortical) arousal index (ArI) and the AHI from full PSG recordings based on these Somnolyzer scorings.

### 2.3 Detection of autonomic arousals using artificial intelligence

**FIGURE 1:**
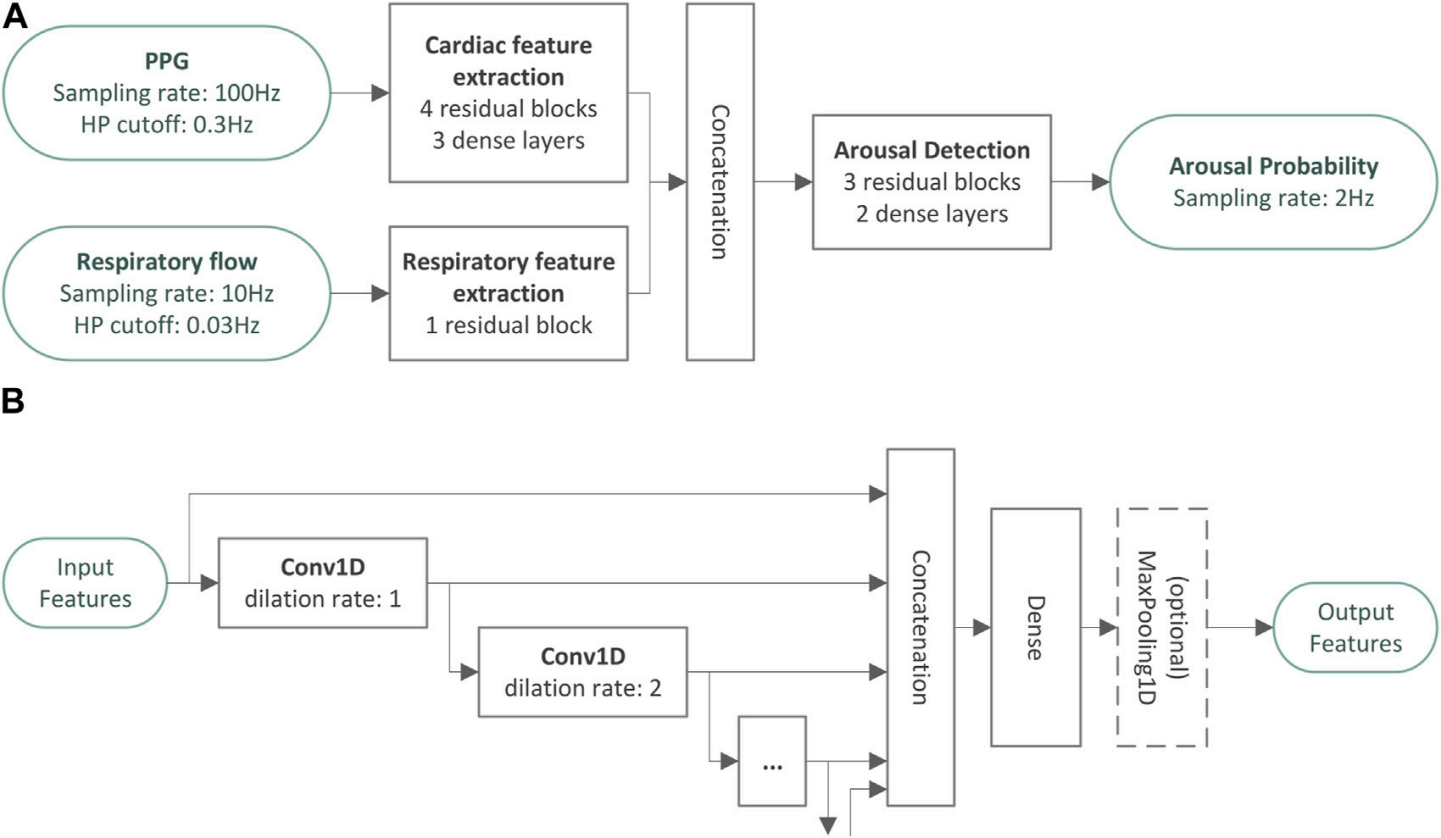
Architecture overview of the autonomic arousal detection model. Panel **(A)** provides an overview over the architecture of the autonomic arousal detection model developed in the present study. There are two feature extraction modules that extract cardiac features from the photoplethysmography signal and respiratory features from the respiratory flow signal. The cardiac and respiratory features are combined and used as input for the final arousal detection module which generates an arousal probability sampled at 2 Hz. All parts of the model use residual convolutional network blocks as illustrated in panel **(B)** to successively increase the feature complexity while reducing temporal resolution.

A deep convolutional neural network was developed to detect autonomic arousals using the raw PPG signal and the respiratory flow signal as inputs. The model consists of three modules that are illustrated in Figure 1A. All parts of the model use residual convolutional network blocks as illustrated in Figure 1B, which successively increase the feature complexity while reducing temporal resolution.

The design of the residual convolutional blocks was inspired by the encoder portion of the atrous spatial pyramid pooling scheme presented in Chen et al. (2017). One-dimensional convolutions extract local temporal context information to enrich the extracted features for each point in time. The dilation rate of a convolutional layer controls how many input time steps are skipped by this layer to allow for sparser representations of a larger temporal context. For example, a dilation rate of two implies that the convolutional layer only looks at every second sampling point. In the residual convolutional blocks of our model, multiple one-dimensional convolutions with small kernels and exponentially increasing dilation rates were stacked to extract features from a large temporal context with a relatively small set of network parameters. Finally, a dense layer was used to create a more compact representation of these features, and a maximum pooling layer reduces the temporal resolution. We stacked residual convolutional blocks in each module to convert the raw input signals to rich feature sets sampled at the desired output sampling rate (i.e., 2 Hz).

The first module consists of a cardiac feature extraction module and comprises four residual convolutional blocks followed by three dense layers, developed to estimate instantaneous heart rate (IHR) at a sampling rate of 2 Hz. In the present study, we used PPG as input, resampled to 100 Hz and pre-processed with a 0.3 Hz high pass filter. The second module comprises a single residual block which uses the respiratory flow resampled at 10 Hz and high pass filtered with a cut-off frequency of 0.03 Hz as input. Both feature extraction modules produce feature sets sampled at 2 Hz. The final arousal detection module comprises 3 residual blocks without any further temporal pooling to produce features also sampled at 2 Hz. Finally, 2 dense layers are used to convert the features into a single arousal event probability with values between 0 and 1. The entire network uses a temporal context of approximately ±45 s to determine the arousal probability at each point in time.

First, the cardiac feature extraction module was trained separately with an additional data augmentation step, where inputs were temporally stretched and squeezed within a predefined range to cover a wide range of possible heart rates. The target for training this module consisted of IHR derived from parallelly recorded ECG data from the full PSG using the R-peak localization algorithm developed by Fonseca et al. (2014). Although we used PPG in this study, we deliberately chose to use IHR as the only cardiac feature in the arousal detection model in order for the final algorithm to be agnostic regarding the actual cardiac input signal, which can also be ECG or any other cardiac sensor modality. Afterwards, the entire model was trained end-to-end using cortical arousals as the target. During this second training phase, the parameters of the cardiac feature extraction module obtained in the initial training phase were fixed. The training targets were sequences sampled at 2 Hz containing the scorings of cortical arousals.

The output of the model can be interpreted as a continuous arousal probability sampled at 2 Hz (i.e., a temporal resolution of 0.5 s). Consecutive sequences of output samples where the arousal probability exceeded a threshold for at least 2 s were considered as part of (individual) autonomic arousal events.

The dataset contained only one recording per subject and was split into four folds containing 25% of the subjects of each database. Subject selection was performed using pseudo-random sampling without replacement, using a fixed seed to guarantee reproducibility. Four-fold cross-validation was performed by iteratively and sequentially combining three folds as training set—used to train the neural network—and evaluating its performance on the data of the remaining validation fold, which was never used in the same iteration to fit, tune or otherwise adjust any parameter of the model for that iteration. The threshold used to detect autonomic arousal events from the output probabilities was chosen individually for each of the four models (one per iteration) by maximizing the F1-score for the detection of arousals on the corresponding training set.

### 2.4 Scoring of sleep and autonomic arousals in HSAT recordings

In HSAT recordings, we used the clinically validated cardio-respiratory sleep staging (CReSS) algorithm as published in Bakker et al. (2021) to infer sleep stages and total sleep time. Autonomic arousals were detected using our novel artificial neural network. Using cross-validation as described, autonomic arousals were always scored with the model that had been trained without any data from the current subject (i.e., when that subject was part of the validation fold). Respiratory events were also detected using the Somnolyzer auto-scoring algorithm.

We calculated three different HSAT-derived surrogates for the AHI. The first estimate corresponds to the traditionally reported respiratory event index (REI_HSAT_), which uses the entire monitoring or recording time as its denominator and does not consider autonomic arousals for the confirmation of hypopneas. As such, the respiratory event index was calculated as the number of apneas and hypopneas (confirmed by oxygen desaturations ≥3%) scored per hour of recording time. The second estimate (AHI_CReSS_) uses sleep stages estimated by the CReSS algorithm to filter respiratory events that were scored during wakefulness: it uses the accurate estimate of total sleep time derived from the cardio-respiratory sleep staging and was calculated as the number of apneas and hypopneas (confirmed by oxygen desaturations ≥3%) scored during sleep, per hour of sleep as determined by CReSS. Finally, the third estimate (AHI_CReSS+AutAr_) considers the CReSS-derived sleep-wake information and additionally uses autonomic arousals to score hypopneas: it was scored as the number of apneas and hypopneas (confirmed by oxygen desaturations ≥3% or autonomic arousals) scored during sleep per hour of sleep as determined by CReSS.

### 2.5 Events in wakefulness

Events during wakefulness should not be considered for the calculation of event indices. Apneas and hypopneas were counted if they overlapped with a sleep epoch. We considered cortical and autonomic arousals if they started during sleep or within the first or last 15 s of a wake period. The tolerance of 15 s was introduced to also capture arousals that led to awakenings.

### 2.6 Statistical analyses

#### 2.6.1 Comparison of the autonomic arousal index

To assess the agreement between the HSAT-derived autonomic arousal index (AutArI) as the number of autonomic arousals per hour of sleep as determined by CReSS and the arousal index (ArI) derived from the PSG, we generated scatter plots, calculated the intraclass correlation coefficient (ICC) of absolute agreement with its 95% confidence interval, generated Bland-Altman plots, and calculated the bias and levels of agreement with 95% confidence intervals. Furthermore, we replicated the receiver-operating characteristic (ROC) analysis used by Pillar et al. (2002) and generated a precision-recall curve to assess the autonomic arousal index’s performance in detecting a pathological ArI ≥20 by varying the threshold for the autonomic arousal index.

#### 2.6.2 Comparison of the apnea-hypopnea index

To compare HSAT-derived surrogate measures to the ground-truth AHI_PSG_, we generated scatter plots and calculated the intraclass correlation coefficient (ICC) of absolute agreement with 95% confidence intervals (Koo and Li, 2016). Furthermore, we generated Bland-Altman plots and calculated the bias and levels of agreement with 95% confidence intervals (Bland and Altman, 1999).

#### 2.6.3 Evaluation of SDB classification performance

To demonstrate the effects of using different AHI surrogates, we provided confusion tables for the categorization of SDB into None (AHI <5), Mild (5≤ AHI <15), Moderate (15 ≤ AHI < 30), and Severe (AHI ≥30). We calculated accuracy, sensitivity, specificity, positive likelihood ratio (LR+), negative likelihood ratio (LR-), and the Cohen’s *κ* for detecting an AHI greater than or equal to thresholds of 5, 15, or 30. Furthermore, we calculated the Cohen’s *κ*, the accuracy for all 4 classes combined, and the fraction of subjects where SDB severity was under- or overestimated. We tested for statistically significant differences between the performance of the AHI_CReSS_ and the REI, as well as between the AHI_CReSS+AutAR_ and the REI. Following the guidelines for statistical testing in clinical trials presented by Kishore and Mahajan (2020), we used two-sided 95% confidence intervals and *p*-values to determine which metrics differed significantly. For binomial proportions, confidence intervals were estimated (accuracy, sensitivity, specificity, fraction of under- or overestimated SDB severity) using the Wilson Score interval with continuity correction (Newcombe, 1998). We used the method proposed in Simel et al. (1991) to estimate confidence intervals for likelihood ratios. The formula for estimating confidence intervals for Cohen’s *κ* coefficients was provided by Cohen himself (Cohen, 1960). Given an acceptable false detection rate of 5%, we applied the Benjamini-Yekutieli procedure to all tests regarding the AHI_CReSS_ as well as to all tests regarding the AHI_CReSS+AutAr_ to control the false discovery rate in multiple tests under arbitrary dependency (Benjamini and Yekutieli, 2001). The procedure resulted in thresholds of 0.0012 and 0.0062 for *p*-values for tests regarding the AHI_CReSS_ and the AHI_CReSS+AutAr_, respectively.

## 3 Results

A total number of 245 subjects were included in this study. Table 1 summarizes the demographic information and the distribution of SDB severity for the two databases. Furthermore, the distributions of the obstructive apnea index (OAI) and the central apnea index (CAI) as the number of obstructive or central apneas per hour of sleep is provided to illustrate that obstructive and central apnea patients are both well represented in the data. Further information about clinical co morbidities of the patients was not available.

**TABLE 1:** Demographic data for the databases used in the cross-validation.

	Somnoval	Somnapatch
Included subjects	97	148
Age [mean ± std, (min, max)]	57.0 ± 14.0 [22, 81]	53.9 ± 13.0 [23, 80]
Sex (m, f)	60 (62%) m, 36 (37%) f	83 (56%) m, 65 (44%) f
BMI [mean ± std, (min, max)]	—	30.1 ± 5.9 [18.3, 55.2]
ArI_PSG_ [mean ± std, (min, max)]	24.5 ± 13.3 [5.3, 75.0]	25.5 ± 18.0 [1.3, 98.4]
AHI_PSG_ [mean ± std, (min, max)]	26.8 ± 20.7 [0.2, 84.6]	32.3 ± 25.5 [0.9, 111.2]
No SDB (AHI <5)	13 (13.4%)	17 (11.5%)
Mild SDB (5 ≤ AHI <15)	21 (21.6%)	24 (16.2%)
Moderate SDB (15 ≤ AHI <30)	23 (23.7%)	41 (27.7%)
Severe SDB (30 ≤ AHI)	40 (41.2%)	66 (44.6%)
OAI_PSG_ [mean ± std, (min, max)]	6.7 ± 9.5 [0.0, 44.2]	20.4 ± 21.6 [0.0, 128.0]
CAI_PSG_ [mean ± std, (min, max)]	2.5 ± 6.4 [0.0, 39.2]	4.1 ± 9.2 [0.0, 71.1]

Included subjects and distributions of age, sex (m: male, f: female), body mass index (BMI), cortical arousal index (ArI_PSG_), apnea-hypopnea index (AHI_PSG_), the severity of sleep disordered breathing (SDB) according to the full polysomnographic recordings, and distributions of obstructive apnea index (OAI_PSG_) and central apnea index (CAI_PSG_). For continuous variables, the table reports the mean and standard deviation as well as the extreme values in square brackets. For categorical data, counts and percentages are reported. In the Somnoval database the BMI, was not collected, and the sex was not recorded for one subject.

### 3.1 Comparison of the autonomic arousal index

**FIGURE 2:**
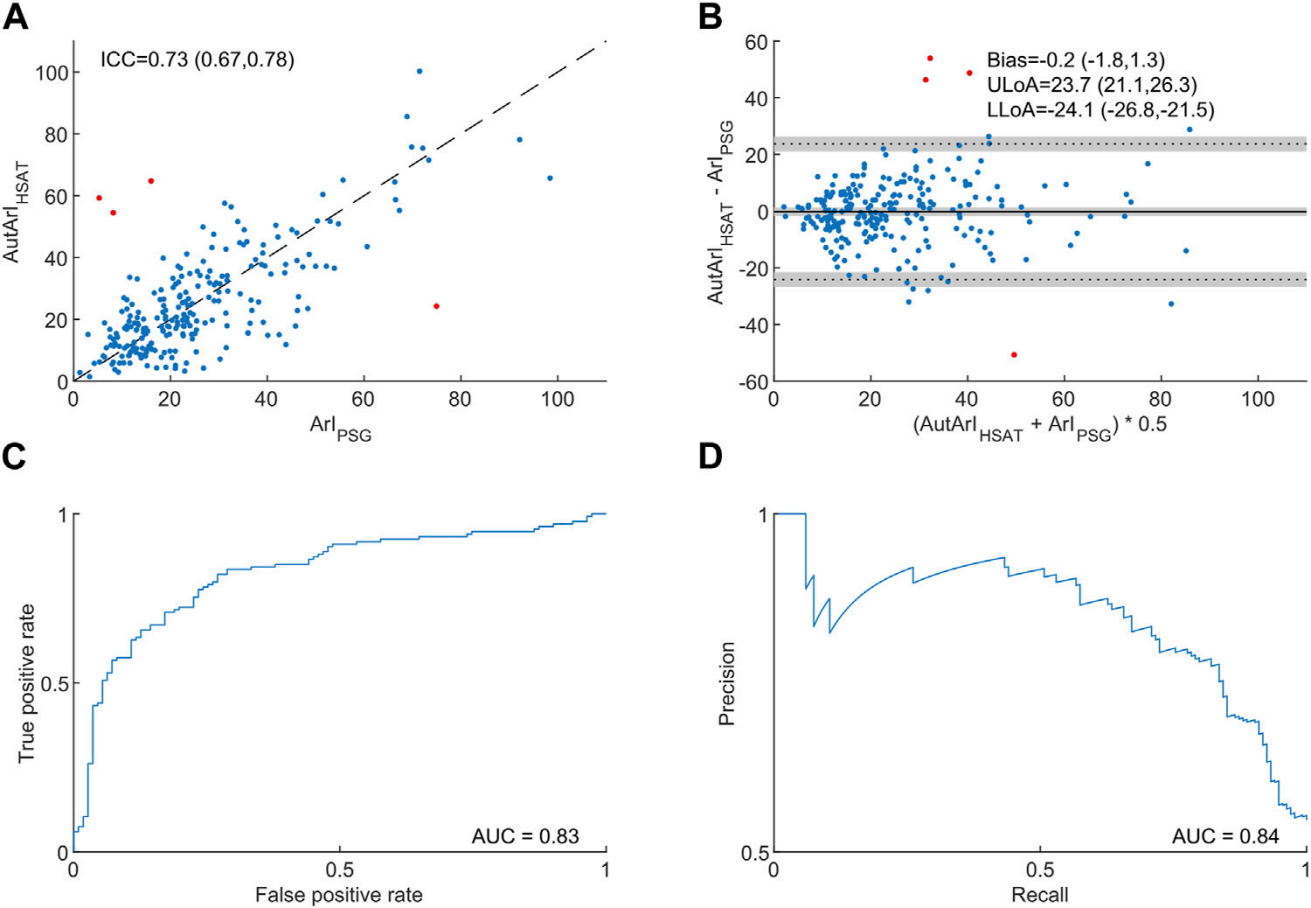
Comparison of the autonomic arousal index derived from home sleep apnea tests to the arousal index from polysomnography. Panel **(A)** shows a scatter plot to compare the autonomic arousal index (AutArI_HSAT_) to the cortical arousal index (ArI_PSG_) and the intraclass correlation coefficient of absolute agreement with a 95% confidence interval in brackets. Panel **(B)** illustrates differences between the AutArI_HSAT_ and the ArI_PSG_ in a Bland-Altman plot and provides bias and levels of agreement together with their respective 95% confidence intervals in brackets. Panel **(C)** shows the autonomic arousal index’s receiver operating characteristic (ROC) curve and the area under the curve for detecting an arousal index ≥20. Panel **(D)** shows the autonomic arousal index’s precision-recall curve and the area und the curve for detection an arousal index ≥20. We identified four outliers and highlighted them in red (see the discussion section for information about the outliers).

Figure 2 compares the HSAT-derived autonomic arousal index (AutArI) to the gold-standard arousal index (ArI) derived from PSG. The Intra-class correlation coefficient was 0.73 with a 95% confidence interval of (0.67, 0.78) and a bias in the estimation of the arousal index of −0.2 events/hour with a 95% CI of (−1.8, 1.3). The levels of agreement and their respective 95% confidence intervals were −24.1 (−26.8, −21.5) and 23.7 (21.1, 26.3) events/hour. We assessed the autonomic arousal index’s diagnostic ability for detecting an ArI ≥20 (134 out of the 245 subjects in the database) with a receiver-operating characteristic (ROC) curve, achieving an area under the ROC curve of 0.83, and a precision-recall (PR) curve, achieving an area under the PR curve of 0.84.

### 3.2 Comparison of the apnea-hypopnea index

**FIGURE 3:**
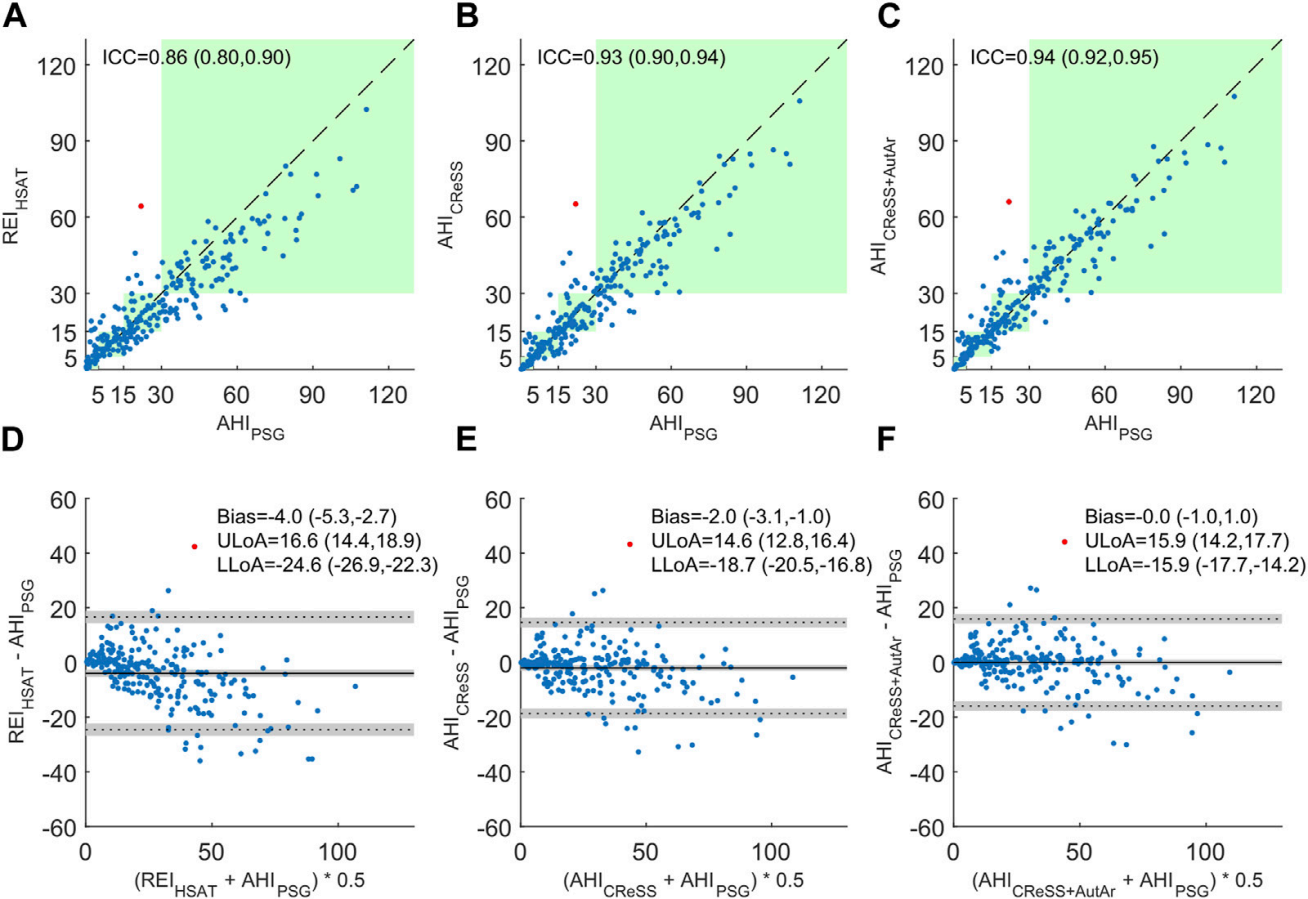
Comparison of respiratory event indices derived from home sleep apnea tests to the apnea-hypopnea index derived from polysomnography. Panels **(A–C)** show scatter plots of the respiratory event index as the number of apneas and hypopneas events per hour of monitoring time (REI_HSAT_), the number of apneas and hypopneas per hour of total sleep time derived from cardio-respiratory signals (AHI_CReSS_), and the number of apneas and hypopneas per hour of total sleep time derived from cardio-respiratory signals where autonomic arousals were used to confirm hypopneas (AHI_CReSS+AutAr_), as compared to the AHI_PSG_. The respective intraclass correlation coefficients of absolute agreement (ICC) are provided and their respective 95% confidence intervals displayed in brackets. The green background color indicates areas where both, the surrogate and the gold-standard measurement would yield the same severity classification using thresholds of 5, 15, and 30. Panels **(D–F)** show Bland-Altman plots to illustrate differences between the surrogate measurements REI_HSAT_, AHI_CReSS_, and the AHI_CReSS+AutAr_ and the gold-standard AHI_PSG_. The bias and levels of agreement for the surrogate measures are provided with their respective 95% confidence intervals indicated in brackets. One outlier was identified and highlighted in red (see the discussion section for information about the outlier).

Figure 3 illustrates the comparison of HSAT-derived surrogates to the gold standard AHI_PSG_ for all 245 studies. Using the respiratory event index reflecting the number of events per hour of monitoring time (REI_HSAT_) resulted in an intra-class correlation coefficient of 0.86 with a 95% confidence interval (CI) of (0.80, 0.90) and a mean difference (bias) of −4.0 events/hour with a 95% CI of (−5.3, −2.7). When cardio-respiratory staging was used to estimate total sleep time and to remove false positive events during wakefulness (AHI_CReSS_), the ICC improved to 0.93 with a 95% CI of (0.90, 0.94) and the bias to −2.0 events/hour with a 95% CI of (−3.1, −1.0). Finally, by additionally scoring autonomic arousals and confirming hypopneas associated with them (AHI_CReSS+AutAr_), the ICC was further improved to 0.94 with a 95% CI of (0.92, 0.95), and the bias was reduced to 0.0 with a 95% CI of (−1.0, 1.0). With each improvement of the index estimation, the levels of agreement for the differences also come closer to the bias. Furthermore, the Bland-Altman plot in panel (D) comparing the REI_HSAT_ to the AHI_PSG_ clearly shows an underestimation proportional to the severity, which is much less pronounced or even absent in panels (E) and (F) comparing the AHI_CReSS_ and the AHI_CReSS+AutAr_ to the AHI_PSG_.

### 3.3 Evaluation of SDB classification performance

Table 2 compares the three AHI surrogates with respect to their ability to assess the severity of sleep disordered breathing. We generated confusion tables for the three index estimations comparing the resulting severity classification into no, mild, moderate, and severe SDB (using thresholds of 5, 15, and 30) to the ground-truth derived from the full PSG (Epstein et al., 2009). The diagonals of the matrices (blue) show the number of correct classifications, while underestimations of SDB severity are found above the diagonal, and overestimations are located below the diagonal. Overall, using the REI_HSAT_ resulted in the correct severity classification of 172 subjects (70.2%, *κ* = 0.58), while severity was underestimated in 46 subjects (18.8%) and overestimated in 27 subjects (11.0%). After applying cardio-respiratory sleep staging and using the AHI_CReSS_ to estimate severity, 189 subjects (77.1%, *κ* = 0.67) were classified correctly, and the severity was underestimated in 32 subjects (13.1%) and overestimated in 24 subjects (9.8%). Finally, when hypopneas were scored with autonomic arousals and the AHI_CReSS+AutAr_ was used to assess SDB severity, the number of correctly classified subjects increased to 197 (80.4%, *κ* = 0.72) and the number of under-diagnosed subjects was further reduced to 18 (7.3%). The number of subjects with overestimated SDB severity slightly increased to 30 (12.2%). It is worth mentioning that for all three AHI surrogates, most misclassifications were between adjacent severity categories. The number of subjects where the severity was under- or overestimated by more than one severity category was 4 (1.6%), 0, and 1 (0.4%) when using the REI_HSAT_, AHI_CReSS_ and AHI_CReSS+AutAr_, respectively.

**TABLE 2:** Confusion tables for the classification performance of respiratory event indices derived from home sleep apnea tests.

			True class			
			No	Mild	Moderate	Severe
			AHI_PSG_<5	5≤ AHI_PSG_<15	15≤ AHI_PSG_<30	AHI_PSG_≥30
Predicted class	REI_HSAT_	No	16 (53%)	0 (0%)	0 (0%)	0 (0%)
		Mild	10 (33%)	38 (84%)	20 (31%)	0 (0%)
		Moderate	4 (13%)	7 (16%)	38 (59%)	26 (25%)
		Severe	0 (0%)	0 (0%)	6 (9%)	80 (75%)
	AHI_CReSS_	No	22 (73%)	4 (9%)	0 (0%)	0 (0%)
		Mild	8 (27%)	34 (76%)	13 (20%)	0 (0%)
		Moderate	0 (0%)	7 (16%)	42 (66%)	15 (14%)
		Severe	0 (0%)	0 (0%)	9 (14%)	91 (86%)
	AHI_CReSS+AutAr_	No	20 (67%)	2 (4%)	0 (0%)	0 (0%)
		Mild	10 (33%)	35 (78%)	9 (14%)	0 (0%)
		Moderate	0 (0%)	7 (16%)	43 (67%)	7 (7%)
		Severe	0 (0%)	1 (2%)	12 (19%)	99 (93%)

Sleep disordered breathing (SDB) was classified into None, Mild, Moderate, and Severe by applying thresholds of 5, 15, and 30 to the apnea-hypopnea index derived from polysomnography (AHI_PSG_) and its surrogates derived from home sleep apnea test (HSAT) data: the number of apneas and hypopneas per hour of monitoring time (REI_HSAT_), the number of apneas and hypopneas per hour of total sleep time derived from cardio-respiratory signals (AHI_CReSS_), and the number of apneas and hypopneas per hour of total sleep time derived from cardio-respiratory signals where hypopneas were also confirmed with autonomic arousals (AHI_CReSS+AutAr_). Columns correspond to the true class as obtained from the PSG_AHI_, and rows correspond to the predicted class using HSAT-derived surrogates. The cells contain absolute counts and percentages of matching and mismatching classifications.

Table 3 summarizes performance measures and provides 95% confidence intervals for the binary classification tasks of determining an AHI greater or equal than the diagnostic thresholds of 5, 15, and 30 and presents overall performance statistics with 95% confidence intervals for the four-class classification problem into none, mild, moderate, and severe SDB. For the detection of an AHI ≥5, the introduction of CReSS led to statistically significant (but clinically irrelevant) changes in the sensitivity and in the negative likelihood ratio: the sensitivity was reduced from 1.0 to 0.99, and the negative likelihood ratio increased from 0.0 to 0.01. For an AHI ≥15, the combination of cardio-respiratory sleep staging and autonomic arousal detection yielded statistically significant improvements for the accuracy, which increased from 0.873 to 0.931; the sensitivity which increased from 0.882 to 0.947; and the Cohen’s *κ*, which increased from 0.712 to 0.837. The specificity and the positive and negative likelihood ratios improved as well, but these changes were not statistically significant. For the detection of an AHI ≥30, the introduction of CReSS and the hypopnea scoring using autonomic arousals led to statistically significant improvements in sensitivity which increased from 0.755 to 0.934; the negative likelihood ratio that decreased from 0.256 to 0.073; and the Cohen’s *κ*, which increased from 0.728 to 0.835. The accuracy improved as well, while the specificity and the positive likelihood ratio were both reduced. However, these changes were not statistically significant.

**TABLE 3:** Comparison of diagnostic performance for different event indices derived from home sleep apnea tests.

Task	Prev	Metric	REI_HSAT_	AHI_CReSS_	P	AHI_CReSS+AutAr_	P
AHI ≥5	0.878	Acc	0.943 (0.90, 0.96)	0.951 (0.91, 0.97)	0.705	0.951 (0.91, 0.97)	0.705
		Sens	1.000 (0.98, 1.00)	0.981 (0.95, 0.99)	<0.001	0.991 (0.96, 1.00)	<0.001
		Spec	0.533 (0.35, 0.68)	0.733 (0.54, 0.85)	0.041	0.667 (0.47, 0.80)	0.199
		LR+	2.143 (1.46, 3.14)	3.680 (2.03, 6.66)	0.074	2.972 (1.79, 4.93)	0.205
		LR-	0.000 (0.00, —)	0.025 (0.01, 0.07)	<0.001	0.014 (0.00, 0.06)	<0.001
		κ	0.667 (0.50, 0.84)	0.758 (0.63, 0.89)	0.182	0.743 (0.60, 0.89)	0.299
AHI ≥15	0.694	Acc	0.873 (0.82, 0.91)	0.918 (0.88, 0.95)	0.035	0.931 (0.89, 0.96)	0.006
		Sens	0.882 (0.82, 0.92)	0.924 (0.87, 0.95)	0.110	0.947 (0.90, 0.97)	0.007
		Spec	0.853 (0.75, 0.91)	0.907 (0.81, 0.95)	0.247	0.893 (0.80, 0.94)	0.422
		LR+	6.02 (3.48, 10.4)	9.90 (4.88, 20.1)	0.168	8.88 (4.61, 17.1)	0.245
		LR-	0.138 (0.09, 0.21)	0.084 (0.05, 0.14)	0.068	0.059 (0.03, 0.11)	0.010
		κ	0.712 (0.62, 0.81)	0.812 (0.73, 0.89)	0.013	0.837 (0.76, 0.91)	<0.001
AHI ≥30	0.433	Acc	0.869 (0.82, 0.90)	0.902 (0.86, 0.93)	0.147	0.918 (0.88, 0.95)	0.022
		Sens	0.755 (0.66, 0.82)	0.858 (0.77, 0.91)	0.013	0.934 (0.86, 0.96)	<0.001
		Spec	0.957 (0.90, 0.98)	0.935 (0.88, 0.96)	0.296	0.906 (0.84, 0.94)	0.015
		LR+	17.5 (7.93, 38.5)	13.3 (7.02, 25.1)	0.394	9.99 (5.94, 16.8)	0.035
		LR-	0.256 (0.18, 0.36)	0.151 (0.09, 0.24)	0.028	0.073 (0.04, 0.15)	0.001
		κ	0.728 (0.64, 0.82)	0.799 (0.72, 0.88)	0.067	0.835 (0.77, 0.90)	0.003

Diagnostic performance for binary classification tasks at thresholds of 5, 15, and 30 for the apnea-hypopnea index (AHI). For each diagnostic threshold, the table shows the prevalence (Prev.) within our dataset. Accuracy (Acc), sensitivity (Sens), specificity (Spec), positive likelihood ratio (LR+), negative likelihood ratio (LR-), and Cohen’s *κ* (*κ*) for AHI, surrogates derived from HSAT, are listed. Point estimates and two-sided 95% confidence intervals are compared in three columns for the number of apneas and hypopneas per hour of monitoring time (REI_HSAT_), per hour of total sleep time derived from cardio-respiratory signals (AHI_CReSS_), and per hour of total sleep time derived from cardio-respiratory signals where hypopneas were also confirmed with autonomic arousals (AHI_CReSS+AutAr_). Metrics for the AHI_CReSS_, and AHI_CReSS+AutAr_ were tested for statistically significant differences to the REI, and corresponding *p*-values are provided in the columns next to the point estimates and confidence intervals. Statistically significant results are highlighted in grey.

Table 4 presents performance metrics for the four-class classification into severity categories. By adding sleep and arousal information derived from autonomic features, significant improvements could be achieved regarding the overall accuracy with an improvement from 0.702 to 0.804, the Cohen’s *κ* which increased from 0.58 to 0.716, as well as the fraction of subjects where SDB severity was underestimated which was reduced from 0.188 to 0.073. There were no significant changes in the fraction of subjects where SDB was overestimated.

**TABLE 4:** Comparison of the severity classification performance for different event indices derived from home sleep apnea tests.

Metric	REI_HSAT_	AHI_CReSS_	P	AHI_CReSS+AutAr_	P
Accuracy	0.702 (0.64, 0.75)	0.771 (0.71, 0.82)	0.019	0.804 (0.75, 0.85)	<0.001
Underestimated	0.188 (0.14, 0.24)	0.131 (0.09, 0.18)	0.022	0.073 (0.05, 0.11)	<0.001
Overestimated	0.110 (0.08, 0.15)	0.098 (0.07, 0.14)	0.624	0.122 (0.09, 0.17)	0.596
Cohen’s *κ*	0.580 (0.50, 0.66)	0.674 (0.60, 0.75)	0.014	0.716 (0.64, 0.79)	<0.001

Diagnostic performance for the four-class classification task of categorizing sleep disordered breathing into no, mild, moderate, and severe using thresholds of 5, 15, and 30. Accuracy, the fraction of subjects where the severity was underestimated, the fraction of subjects where the severity was overestimated, and Cohen’s *κ* for AHI, surrogates derived from HSAT, are listed. Point estimates and two-sided 95% confidence intervals are compared in three columns for the number of apneas and hypopneas per hour of monitoring time (REI_HSAT_), per hour of total sleep time derived from cardio-respiratory signals (AHI_CReSS_), and per hour of total sleep time derived from cardio-respiratory signals where hypopneas were also confirmed with autonomic arousals (AHI_CReSS+AutAr_). Metrics for the AHI_CReSS_, and AHI_CReSS+AutAr_ were tested for statistically significant differences to the REI, and corresponding *p*-values are provided in the columns next to the point estimates and confidence intervals. Statistically significant results are highlighted in grey.

## 4 Discussion

In this work, we showed that by extracting surrogate sleep and arousal information from the signals obtained by HSATs, significant improvements can be made in the estimation of the AHI. We used cardio-respiratory sleep staging and assessed autonomic arousals in order to mimic the scoring rules recommended for PSG in PG recordings. Consequently, the ICC of absolute agreement between the HSAT-derived surrogate and the gold standard of PSG-derived AHI increased to 0.94 with a 95% confidence interval of (0.92, 0.95). This indicates excellent agreement that is in the same range as the inter-scorer agreement between manual scorers annotating gold-standard PSGs (Koo and Li, 2016).

Using recommended hypopnea scoring rules, manual experts were reported to agree on the AHI with a bias of less than ±1.5 and a 95% limits of agreement of approximately ±18 to 22 events per hour of sleep when scoring full PSG studies (Massie et al., 2018; Van Pee et al., 2022). We observed a bias of −4 events/hour when using the REI, confirming our initial hypothesis that PG can and often does underestimate SDB severity. This bias could be eliminated by adding cardio-respiratory sleep staging to determine the total sleep time and confirming hypopneas not only by desaturations, but also by autonomic arousals. Moreover, the limits of agreement were narrowed down to less than ±16 events per hour of sleep. As expected, the agreement between the HSAT-derived REI and the PSG-derived AHI was below typical inter-scorer reliability reported for the AHI. Adding sleep and arousal estimations from the autonomic nervous system raised the agreement between PG and PSG to the same level as observed between manual scorers scoring full PSG recordings (Massie et al., 2018; Van Pee et al., 2022), suggesting an improvement on diagnostic accuracy to a level suitable for clinical applications.

Regarding the binary classification at diagnostic thresholds of 5, 15 and 30, we expected an increase in sensitivity for all three thresholds, possibly at the cost of small losses in specificity, contributing to an overall increase in accuracy. The specificity for detecting an AHI ≥5 increased substantially from 53.3% to 73.3% when introducing CReSS-derived total sleep time in the AHI calculation. At first, this might seem counter-intuitive because the reduction of the denominator in the AHI calculation from monitoring time to total sleep time should increase the estimated AHI and therefore increase diagnostic sensitivity. A closer look at the scorings revealed that some of the false positives (REI ≥5 but AHI <5) were caused by respiratory events detected during wakefulness, possibly caused by motion artifacts. CReSS excluded these respiratory disturbances during wakefulness, effectively increasing the specificity of the HSAT devices without any relevant loss in sensitivity. For the task of detecting an AHI ≥15, the introduction of CReSS increased sensitivity and specificity, and therefore also the accuracy from 87.3% to 91.8%. Sensitivity and accuracy were increased further by confirming hypopneas with autonomic arousals such that the final accuracy was 93.1% with a sensitivity of 94.7% and a specificity of 89.3%. Again, sensitivity and specificity both clearly improved when adding the sleep and arousal information to the HSAT recordings. Finally, for an AHI ≥30, sensitivity increased significantly from 75.5% to 93.4% while the specificity remained above 90% and the accuracy improved from 86.9% to 91.8%.

Van Pee et al. (2022) reported interscorer agreement between manual scorings of the AHI in full PSGs of 93% (*κ* = 0.69), 90% (*κ* = 0.79), and 92% (*κ* = 0.83) at diagnostic thresholds of 5, 15, and 30, respectively. From the confusion tables presented by Massie et al. (2018), we can easily derive very similar accuracies and Cohen’s *κ* values for the interscorer agreement of 93% (*κ* = 0.68), 91% (*κ* = 0.81), and 89% (*κ* = 0.76) for the respective diagnostic thresholds. When comparing these values to our results, it became clear that the classification performance with REI was only comparable to the manual scoring of full PSGs for a diagnostic threshold of the AHI being greater or equal to 5. When adding sleep and arousal estimations from the autonomic nervous system to the HSAT analysis, the SDB classification performance improved to the same level as observed between manual experts scoring full PSG recordings with accuracies of 95% (*κ* = 0.74), 93% (*κ* = 0.84), and 92% (*κ* = 0.84) at the respective thresholds. When classifying the SDB severity into four classes, Massie et al. (2018) reported an interscorer agreement on full PSGs of 81% with a Cohen’s *κ* of 0.74, and Van Pee et al. (2022) reported an interscorer agreement of 77% with a Cohen’s *κ* of 0.66. As can be seen in Table 4, using the REI as surrogate for the AHI_PSG_ clearly fell below the reported inter-scorer agreement. However, by adding cardio-respiratory sleep staging and autonomic arousal information to the polygraphies, classification performance increased to the level of interscorer agreement amongst manual experts scoring full PSG recordings. Using the combination of sleep and arousal information derived from cardio-respiratory signals, the overall classification performance was significantly improved regarding the overall accuracy, Cohen’s *κ*, and the percentage of participants where SDB was underestimated. At the same time, the increase of over-diagnosed patients from 11% to 12.2% was neither statistically significant (*p* = 0.596) nor clinically relevant. These results confirm that using the AHI_CReSS+AutAr_ to assess SDB severity instead of the REI significantly improves the diagnostic sensitivity of HSAT devices, leading to much smaller likelihood of underestimating SDB severity.

In Figure 3, we identified one outlier where the AHI was severely overestimated by all three HSAT-derived estimates. The AHI_PSG_ for this recording was 21.9, while HSAT-derived estimates varied between 64.3 and 66.0. A review of the raw data revealed that this recording contained numerous central apneas during epochs that contained rapid transitions between wake and sleep. The occurrence of sleep transition apnea may yield immediate hyperventilation leading to central apnea upon resumption of sleep (Malhotra and Owens, 2010). This alternating pattern has been described as state instability in previous research (Roberts et al., 2022). In this example, most of these epochs had to be scored as wake, because the EEG data indicated wakefulness for more than half of each 30 s epoch. Consequently, most of these apneas were not considered for the calculation of the AHI_PSG_. This presents a more fundamental limitation of scoring sleep stages in 30-s epochs and could only be fully resolved by adopting, for example, as discussed by Perslev et al. (2021), high-frequency sleep staging, especially for diagnosing a population experiencing sleep apnea. Due to the lack of sleep information, these events were included in the calculation of the REI_HSAT_. The cardio-respiratory sleep staging algorithm scored these transitional epochs as sleep. Therefore, the estimated AHI in this recording remained high even when adding sleep and arousal estimations to the HSAT scoring.

Interscorer agreement for the number of arousals and the arousal index have been reported with intraclass correlation coefficients between 0.52 and 0.80 with outliers as low as 0.09, indicating only limited reliability (Bonnet et al., 2007; Kuna et al., 2013; Magalang et al., 2013; Malhotra et al., 2013; Punjabi et al., 2015). In an early attempt at detecting autonomic arousals from ECG, agreement between cortical and autonomic arousals has been reported with an ICC of 0.19 (Basner et al., 2007). More recently developed algorithms based on PAT or ECG inputs report Pearson’s correlation coefficients between 0.58 and 0.84, depending on the OSA severity distribution of the patients (Pillar et al., 2002; Li et al., 2020). Olsen et al. (2018) report an event-by-event sensitivity of 0.63 and positive predictive value of 0.72. Thus, the agreement between the autonomic arousal index derived from our model and PSG fell within the range of normal interscorer reliability of scoring cortical arousals in a full PSG and is similar to the performance reported for PAT devices detecting autonomic arousals. The ICC between the autonomic arousal index derived from our model (using PPG and respiratory flow as inputs) and the cortical arousal index (derived from neurological signals in the PSGs) was 0.73 with a 95% confidence interval of (0.67, 0.78), indicating moderate to good agreement.

We identified four outliers when comparing the cortical arousal index to the autonomic arousal index (highlighted in Figure 2). In one case a cortical arousal index of 75.0 was heavily underestimated, with an autonomic arousal index of 24.2. A review of the raw data revealed that this patient had severe cardiac arrhythmia which probably reduced the sensitivity of the autonomic arousal detection. Moreover, we identified three cases where relatively low cortical arousal indices of 5.3, 8.2, and 16.0 were overestimated with autonomic arousal indices of 59.2, 54.5, and 64.8. In two of these cases, the autonomic arousals were associated with respiratory events that were not associated with cortical arousals. The third case contained periodic leg movements that were associated with autonomic arousals but not with cortical arousals. Both phenomena have been described previously: Olsen et al. (2018) reported that 81% of autonomic arousals not associated with a matching cortical arousal could be related to respiratory events or leg movements, and Pillar et al. (2002) reported that the agreement between the cortical and autonomic arousal index could be increased significantly when excluding patients with periodic leg movements from the comparison. In our case, the ICC between the autonomic arousal index and the cortical arousal index increased from 0.73 (with 95% CI of 0.67, 0.78) to 0.79 (with 95% CI of 0.74, 0.84) when excluding these four outliers. Although these outliers are exceptions in the overall performance of our method, they also reflect an inherent limitation of this approach: autonomic arousals are not equivalent to cortical arousals and not every cortical arousal coincides with an autonomic arousal, and *vice versa*; and it has been reported how some respiratory events, and especially periodic limb movements, can appear associated with autonomic arousals without a corresponding cortical arousal (Sforza et al., 2000; Sforza et al., 2002; Olsen et al., 2018).

Due to the study design, heart rate information was the only cardiac feature considered for the detection of autonomic arousals. Future studies might provide insights whether the use of additional features extracted from the PPG signal, (i.e., pulse wave amplitudes and waveform or baseline changes) can improve the accuracy of the autonomic arousal detection further. Keeping in mind the growing popularity of wearable devices with PPG sensors (e.g., smart-watches), future research should assess potential improvements of the sleep stage classification and the arousal detection when using these additional PPG-derived features. All data used in this study was recorded in sleep laboratories under supervision. Therefore, the algorithm performance might be slightly overestimated compared to an unsupervised real-world HSAT scenario. However, the relative improvement when comparing the AHI_CReSS+AutAr_ to traditionally used REI should still be similar, because both methods suffer from the same data quality issues when sensors are self-applied, and recordings are not supervised. Our study does not evaluate the algorithm’s performance in the presence of cardiac arrhythmia, autonomic dysfunction, impaired heart rate variability, and medication altering heart rate and respiration rate variability (e.g., beta blockers). Future research is required to assess whether the algorithm performs robustly in these patient populations. Literature, as well as our data, suggest that it is not uncommon that respiratory events or periodic leg movements are associated with autonomic arousals that do not lead to cortical arousals (Olsen et al., 2018). Therefore, future studies could also address the question of whether it might be useful to complement the neurological arousal scoring with the detection of autonomic arousals in full PSGs.

In conclusion, this study is the first to show that adding sleep and arousal information derived from autonomic nervous system activity can improve the diagnostic sensitivity of PGs by significantly reducing the risk of underestimating SDB severity, without a relevant decrease in specificity. In particular, the confirmation of hypopneas using autonomic arousals can raise the sensitivity to a level similar to that of a full PSG recording. The focus of this study was limited to the AHI as the primary tool for assessing SDB severity. Future research could evaluate if similar improvements in accuracy can be achieved for alternative measures relying on respiratory event detection such as hypoxic burden, arousal intensity, or apnea-specific pulse-rate responses (Azarbarzin et al., 2019; Azarbarzin et al., 2021; Malhotra et al., 2021). Another interesting research topic might be to assess how the detection of autonomic arousals might complement the scoring of cortical arousals in the interpretation of PSG. Finally, to fully assess the performance and utility of new devices or methods, a next step also requires validation on independent and publicly available datasets to make the results reproducible and comparable for the scientific community.

## Data Availability

The data analyzed in this study is subject to the following licenses/restrictions: The datasets presented in this article are not readily available because they are property of Philips Sleep and Respiratory Care. Requests to access these datasets should be directed to ES (edmund.shaw@philips.com).
